# Government as Institutional Entrepreneur: Extending Working Life in the UK and Japan^4^

**DOI:** 10.1017/S0047279414000075

**Published:** 2014-07

**Authors:** MATTHEW FLYNN, HEIKE SCHRÖDER, MASA HIGO, ATSUHIRO YAMADA

**Affiliations:** * Centre for Research into the Older Workforce, Newcastle University, Newcastle UK NE1 4SE email: matt.flynn@ncl.ac.uk; ** WU Vienna University of Economics and Business, Welthandelsplatz 1, 1020 Vienna, Austria email: h.s.Schröder@gmail.com; *** Sloan Center on Aging and Work at Boston College, 140 Commonwealth Avenue, Chestnut Hill, MA 02467, USA email: masateru.higo@gmail.com; **** Keio University, 2-15-45 Mita, Minato-ku, Tokyo 108-8345, Japan email: atsuhiro@econ.keio.ac.jp

## Abstract

Through the lens of Institutional Entrepreneurship, this paper discusses how governments use the levers of power afforded through business and welfare systems to affect change in the organisational management of older workers. It does so using national stakeholder interviews in two contrasting economies: the United Kingdom and Japan. Both governments have taken a ‘light-touch’ approach to work and retirement. However, the highly institutionalised Japanese system affords the government greater leverage than that of the liberal UK system in changing employer practices at the workplace level.

## Introduction

This paper discusses institutional regimes surrounding the management of the employment and retirement of older workers in two national contexts: the United Kingdom (UK) and Japan. Due to ageing populations, both nations are facing ageing demographics (OECD, 2011a). Recently, the Organisation for Economic Co-operation and Development (OECD) recommended that member states raise their retirement ages in order to increase the sustainability of existing public pension programmes (OECD, 2005). The dilemmas experienced by governments and employers in these two OECD member states encourage cross-national comparisons of the two countries’ institutional regimes in relation to public policy and employer practices (Doling *et al.*, 2005).

## Methodology

This paper is based on a review of the relevant literature and original interviews conducted by national experts (Bogner *et al.*, 2005; Flick, 2009). The data were from trade unions, government and non-government sources in the two countries. In the UK, these were representatives from the Trades Union Congress (TUC), Chartered Institute of Personnel and Development (CIPD), Age Concern England (ACE), the Age and Employment Network (TAEN), Employers Forum on Age (EFA), the government´s Department for Work and Pensions (DWP) and the Advisory, Conciliation and Arbitration Service (ACAS), a non-departmental public body. Interviews were initially conducted in 2009 at the same time as the Japanese interviews. However, because of a government change the following year, and the fact that the new government made significant changes to public policies in relation to employment, social welfare and public sector occupational pensions, we are also including data drawn from interviews with representatives from these organisations conducted in 2012.

In Japan, we gathered interview data from representatives of the Association for Employment Promotion for the Aged (AEPA), Japan Aging Research Center (JARC), Silver Human Resource Center (SHRC) and the Ministry of Health, Labour and Welfare (MHLW). Interviewees were selected through purposive sampling (Bryman, 2008: 415), and the sample was matched between the two countries.

Interviews were each approximately one hour long, and conducted using semi-structured interview guides exploring public and social policies focusing on work and retirement as well as any developments over time in each national context.

## Institutional entrepreneurship in the UK and Japan

We start with Ebbinghaus’ (2001) discussion of how ‘institutional collusion’ (p. 76) between welfare states and production regimes led, in developed countries, to early exit routes that enabled employers to shed (costly) older workforces. We then examine whether the UK and Japanese governments have taken on the role of ‘institutional entrepreneur’ (IE) (DiMaggio, 1988), or change agent – and if so, how – in seeking to change the balance of this institutional collusion from one favouring early retirement towards one encouraging an extended working life. We argue that the Japanese government has been more effective in intervening in employer human resource management (HRM) practices than the UK government. In the absence of employer intervention, the Japanese government has stepped in directly to offer the older unemployed work opportunities.

In order to conceptually contrast the institutional regimes surrounding age management between the UK and Japan, we utilise institutional theory. The main premise of this theoretical framework is that national institutions significantly influence how organisations design and implement policies and practices (Hall and Soskice, 2001), including HRM (Gooderham *et al.*, 1999; Mesner-Andolsek and Stebe, 2005). Institutions have the direct and indirect function of guiding, regulating or constraining the behaviour of organisations and individual agents. Institutions might be formal, such as laws and regulations, or informal, such as norms of behaviour (North, 1994). Organisations are thought to gain legitimacy and avoid uncertainty in their institutional environment if they comply with such institutions (DiMaggio and Powell, 1991). Our main focus in this paper is to examine the ways in which institutions as such change, and specifically the role of the government and the state in triggering change, rather than how institutions statically influence (organisational) actors and practices in a given institutional context. DiMaggio (1988) proposes that ‘institutional changes’ endogenously arise ‘when organized actors with sufficient resources see in them an opportunity to realize an interest that they value highly’ (p.18). IEs must (a) be party to such divergent institutional change (Eisenstadt, 1980); (b) initiate divergent change that breaks with existing acknowledged institutional templates (Battilana *et al.*, 2009); and (c) have access to the resources, as well as the social position and formal authority, with which to drive institutional change (Battilana, 2006).

According to Evans and Rauch (1999), the state might explicitly propel and ensure economic growth and development by intervening in economic processes – thereby acting as an *entrepreneur*. In particular, the works by Nasra and Dacin (2010) as well as by Child *et al.* (2007) shed light on how the state as a dominant actor can initiate divergent institutional change. Nasra and Dacin (2010) discuss the role of the state, acting as an international entrepreneur, in the development of Dubai from a fishing village into a globalised city, while Child *et al.* (2007) elaborate on the role of the Chinese government in building and promoting an environmental protection system. However, both cases concern national contexts that are, or were at least during the time span covered in each respective research, considered transition economies. Similarly, Battilana *et al*. (2009) argue that most empirical studies on IE have so far been conducted in fields that are emerging, less institutionalised and therefore characterised by higher levels of uncertainty. This is even though they postulate that divergent change might also be initiated and implemented in highly institutionalised fields. As a consequence, they call for a systematic exploration of different levels of institutionalisation in enabling divergent institutional change. In order to do so, they suggest comparative analysis.

We therefore explore, compare and contrast the respective roles of the state, represented by successive governments in the UK and Japan, in initiating and implementing divergent institutional change in the labour market (regulation) with regard to the inclusion of an ageing workforce. We argue that both states and their governments are attempting to take on the role of IE by encouraging and coercing workers and employers to prolong working life beyond the conventional retirement age. For this purpose, the two governments are using the resources and authority derived from the welfare state and employment regulations, and hence ‘agency’, as a way to create and sustain changes in the retirement culture and to therefore move beyond the reproduction of existing institutional structures that would have reinforced the previous early exit paradigm in public policy respectively. Accordingly, we compare the approaches taken, and hence the resources used, by the two countries using two related theoretical frameworks: Esping-Andersen's (1990) *Welfare Capitalism* and Whitley's (1999) *National Business Systems*.

The UK institutional regime has been characterised as a ‘liberal residual’ welfare system (Esping-Andersen, 1990: 69–77) as well as a ‘regulatory regime’ (Whitley, 2008). Employment policy has been guided by an approach to maintain the UK labour market status as ‘the least regulated in Europe’ (Nicoletti *et al.*, 2000: para. 75), taking a ‘light-touch’ (DTI, 2005: para. 6.3.3) approach to regulation in order to foster inward investment. The government has relied more on persuasion than compulsion to change employment practices, demonstrating the business case for doing so (Flynn, 2010).

The Japanese labour market system has been labelled a developmental state, whose role is ‘concerned with setting substantive social and economic goals which involve particular industrial policies’ (Whitley, 1991: 16). Under Japanese law, there is a distinction between *mandatory regulations* and regulations which put a *duty to endeavour* on employers to improve working conditions (Higo and Klassen, 2014). Under the latter, the government promotes good practice and may require employers to draw up action plans, and there are limited penalties for non-compliance. Some aspects of the laws on equality, retirement and work environment carry only *duty to endeavour* provisions. For instance, in the 1987 amendment to the *Labour Standard Law* (1947), the government requested that employers make available flexible work hour options, including *compressed work hours* and *flexitime* options. To date, the adoption of this request has remained largely voluntary for employers, and does not entail any substantial penalty for non-compliance (Higo, 2006). Choi (2012) conceptualises the Japanese welfare system primarily as a ‘productivist’ state, insofar as de-commodification has been a secondary concern to economic growth. Social protection tends to favour those in work, and, in the case of the pension system, those who have been in work for a long time. Kwon (1997) notes that the Japanese welfare state lacks a class-based political underpinning, which is a standard feature of European systems. Rather, the welfare state emerged in ‘piecemeal’ fashion (Wilding, 2000). Esping-Andersen (1997) argues that the Japanese welfare state facilitates strong corporate solidarity, as individual social benefit levels are tied to employers.

There are three contrasting hypotheses which we could set with regard to how the two governments promote a postponement of individuals’ retirement timing. One proposition is that the two governments take a ‘light-touch’ approach to employment regulation (what in the UK is termed ‘business case’ and in Japan is termed ‘duty to endeavour’). Further, welfare provision in both countries is limited, and, as we will explore below, the state in both contexts provides only a small proportion of total pension provision (see OECD, 2011a: 154a).

Second, and in contrast, we might hypothesise that the developmentalist and productivist roles of the Japanese state better enables it to enact major economic and social change, such as raising retirement ages, than does the (to use Whitley's definition) regulatory and safety net UK regime. This is because, in the former country, the state has institutional authority to intervene in employer practices and the welfare state in order to promote economic development. Therefore, although the regulatory and welfare provision are equivalent, we argue that the Japanese government may be a more effective IE in raising the real retirement age.

Third, it might be hypothesised that the UK government has more leeway in triggering institutional change with regard to older workers’ labour market participation as the related institutional field of labour market regulation is less institutionalised than that of Japan. As a consequence, the UK government might have more space to formulate and implement strategies that support the policy goal of extending older workers’ working lives. This is in line with existing research on governments’ capacity to act as IEs, which has thus far focused on less institutionalised fields, such as China and Dubai (Child *et al*., 2007; Nasra and Dacin, 2010.

These three contrasting propositions are based on the assumption that actors’ (in our case the state’s) agency, or rational choice, is constrained by the institutional structures in which such actors live and work (Battilana, 2006). Here we are hypothesising that the two governments, faced with similar age demographic challenges, will take path-dependent public policy approaches that replicate already existing (labour market and welfare state) institutions with regard to old-age employment.

In the following sections, we will discuss older workers’ labour market participation and employer practices in relation to work and retirement in the two countries. We then discuss UK and Japanese public policies relating to age, drawing on secondary literature as well as on our expert interview data, and finally draw conclusions.

## Older workers’ labour force participation

**Figure 1.**
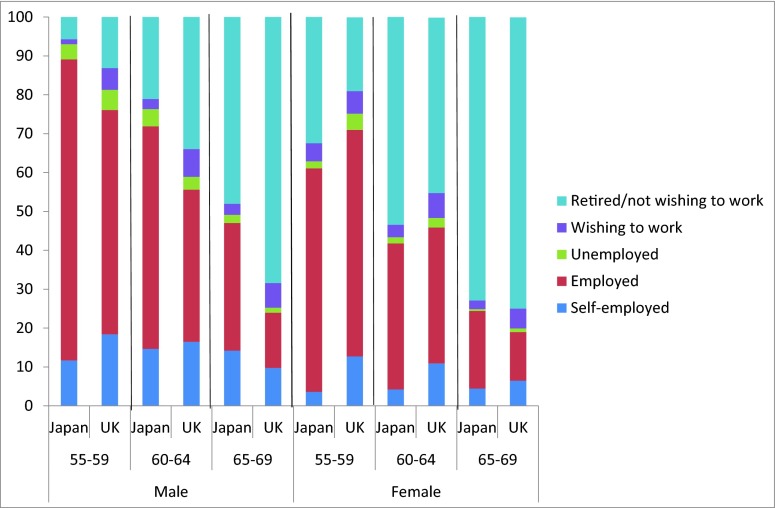
Older people's labour market participation *Source:* UK Labour Force Survey (LFS) October–December 2012 and Japan LFS 2012

Japan has the fifth highest participation rate of older people and, while the UK is lower, it is still higher than the European Union (EU-27) average. Figure 1 presents data extracted from Statistics Bureau Labour Force data (Japan) and Labour Force Survey data (UK). Three differences between the two countries are apparent. First, the Japanese male labour force participation rate is significantly higher than the equivalent UK rate; and the gap widens by age. For women, the proportion of UK women aged fifty-five to sixty-four in employment is higher than in Japan.^1^ However, this reverses from age sixty-five. Second, although more UK workers below sixty-five are self-employed than their Japanese equivalents, Japanese workers are more likely to transition into self-employment at the end of their careers than their UK counterparts. Third, for both men and women, the UK economically inactive individuals are more likely than their Japanese equivalents to want to work. Finally, it should be noted that healthy life expectancy for those who are now sixty-five is higher in Japan than the UK. Unlike work longevity, however, the gap for healthy life expectancy is larger amongst women (age 80.6 in Japan versus age 76.8 in the UK) than men (age 77.6 versus age 75.7) (OECD, 2011b).

Thus, older workers in Japan – men in particular – tend to not only remain in work, but also stay in the labour market longer (i.e., classifying themselves as unemployed or self-employed rather than retired) than their UK equivalents. Although labour market participation for both UK and Japanese older workers (fifty-five to sixty-four years old) is high by OECD standards, among those aged sixty-five and older, the labour force participation rate in Japan remains very high, while that for the UK falls dramatically. This is not to say that UK workers are averse to extending their working lives. One survey found that 80 per cent of people in work would be interested in delaying their retirement timing if they could reduce their workload and working hours during their final years in work (McNair *et al*., 2004). Such ‘phased retirement’ routes are popular but rare (Loretto *et al.*, 2006).

## Public policy

### The UK

Age discrimination is readily accepted in the workplace as intrinsic and is often justified on the grounds of the needs of the business (McNair *et al.*, 2007). Line managers – who often have a central role in shaping their subordinates’ retirement paths – are commonly unaware of, or pay scant attention to, formal HR policies when managing the retirement of subordinates (Flynn, 2010). Policy instruments intended to encourage continued working in later life are usually discretionary, giving managers wide scope for deciding when to apply them (McNair *et al.*, 2007). Compounding this, as McNair (2010) argues, older workers are reluctant to ask for support in extending their working life, either because they consider themselves content with their circumstances or because they expect such requests to be declined.

Against this background, the UK government has sought to encourage employers to retain their older workers, adopting a ‘business case’ approach towards persuading employers to enable older workers to stay economically active longer. The previous Labour government established a campaign unit within the DWP, known as *Age Positive*, to encourage employers to adopt HR practices which facilitate a longer working life. The campaign included research, information and case studies of ‘Age Champions’, employers who had adapted their HR practices to become more age inclusive and had reaped benefits from doing so. The campaign peaked in activity around 2006 when the *Employment Equality (Age) Regulations* were implemented but was wound down by the present Coalition government which deferred to a coalition of stakeholders, including the EFA, known as the Age Action Alliance in employer outreach. According to our DWP interviewee, the government's view was that the state should not lead a campaign intended to change employment practices.

stakeholders are meant to come together with no money and develop and do whatever it is that is needed with whatever minimal involvement they might find useful from civil servants such as myself. (DWP)

Amongst the more prominent Age Champions were civil service departments which had abolished their mandatory retirement agencies five years before having to do so. Public sector pension schemes had also been adapted to both make early retirement more difficult and post-retirement work more profitable (Muller-Camen *et al*., 2011). However, job cuts in the public sector have primarily been met through encouraging older public servants to take early retirement. This has led to a younger workforce profile (NAO, 2012).

Respondents from TAEN and the TUC noted that the government's decision to accelerate the rise in the State Pension Age (SPA) had made the Default Retirement Age (DRA) – which was abolished in 2011 – unsustainable, requiring the government to follow through with its abolition. The representative from CIPD noted that its members (HR managers) who work in organisations which had abolished their own mandatory retirement policies in 2006 reported few disputes with older workers on their retirement plans. She argued that organisational worries about the abolition of the DRA have been ‘over-inflated’, since few older workers expect to stay in work beyond sixty-five.

Despite the DRA abolition, employers are permitted to retain mandatory retirement ages in circumstances in which there was a clear business justification for doing so. Although such cases were expected to be rare and based on job requirements, a 2012 Supreme Court ruling^2^ has created scope for employers to use mandatory retirement as a way to dismiss older workers to make way for younger ones. Since then, a small number of employers, including both Oxford and Cambridge Universities, have reinstated mandatory retirement, justifying them as necessary for retaining younger workers. According to the TUC representative, the relevant trade unions negotiated with Cambridge University to ensure that the mandatory retirement age (which was set at 67 rather than the current SPA) applied only to academics, and required Deans to create phased retirement opportunities for older academics who wanted to continue in work.

State support for the older unemployed and inactive workers who are seeking work has been quite limited and focused exclusively on the low paid and those on state benefits. The previous Labour government had extended its ‘New Deal’ programme to those aged fifty plus. New Deal had initially been set up in 1998 to provide training, subsidised job placements and welfare-to-work policies for people aged eighteen to twenty-four who had been unemployed for over six months. A separate programme for unemployed and inactive people aged fifty plus was introduced in 2000, which provided an employment credit for participants employed for over a year, as well as a £1,500 training allowance. Its success was decidedly modest, with the DWP attributing 167,000 participants as having been supported into work over a six year period (DWP, 2007: 45). The programme was found to be weak in tackling the main problems faced by the older unemployed, particularly age discrimination and the difficulties in transitioning out of declining industries (Kodz and Eccles, 2004). New Deal 50plus has been phased out and replaced with two programmes (Flexible New Deal under the previous Labour government and now the work programme (WP) under the current Coalition government) which make greater use of the private and voluntary sectors in delivering job placements. Although early evidence suggests that job placement programmes targeted at the older unemployed have yielded better results than those focused on the young unemployed (Clayton and Brinkle, 2011), there are concerns within age advocacy groups that WP providers are not adequately meeting the needs of the older unemployed, particularly the long time it takes before an older client can receive support. (Allen, 2012).

Even though the older unemployed are likely to be unemployed longer than their younger counterparts (Fevre, 2011), the UK Jobcentre Plus (JCP) does not offer specific support tailored to older workers. According to a DWP review, JCP advisers tend to favour younger claimants over older ones when making referrals (Kirkpatrick, 2012), partly because the younger unemployed are more likely to hold formal qualifications (Baker, 2009).

The Labour Party, now in opposition, has recently advocated more tailored approaches to supporting the older unemployed back into work through help from career advisors and training vouchers, as well as support from the first month an older person is in receipt of the Job Seeker's Allowance, rather than the current three month threshold. The shadow Work and Pensions Secretary has recently posited Japan as a model for the UK in supporting the older unemployed back into work (Labour Policy Review, 2012).

Finally, changes to state and occupational pension rules have been made to give people over pension age financial incentives to delay labour force exit. Already, the state pension is amongst the most modest in the developed world, replacing 34 per cent of income for an average male earner, compared to a 72 per cent replacement rate across the European Union (OECD, 2011a). Pension law was changed to raise the state pension age to sixty-six by 2020 (DWP, 2006), which the present Coalition government brought forward to 2018 (DWP, 2010). For the state pension, the UK government offers augmentation of annual pension entitlements for those who defer claiming. Furthermore, around 280,000 people aged sixty to sixty-four are drawing state-funded incapacity benefit (IB).^3^ The government has recently introduced more stringent eligibility requirements. According to our DWP informant, the changes to IB rules would have a more immediate impact on older workers’ employability.

Older workers who left work at sixty and expected IB to tide them over now find that they need to find work to 65 and maybe even later. I’m not sure JCP's are ready to support them. (DWP)

### Japan

In order to understand Japanese employers’ perspectives on age management, it is necessary to briefly delineate the institution of *lifetime employment*, which has long characterised the country's labour market and many individuals’ working lives within the country (Higo and Klassen, 2014).

This labour market institutional arrangement underlies today's employer attitudes towards workforce management practices for older workers. It is characterised by two sets of employer HRM practices: (1) Provision of long-term job security for employees and (2) implementation of mandatory retirement rules at the workplace. Together, these two practices have enabled employers to effectively accumulate not only industry-specific but also organisation-specific human capital among their employees (Nomura, 2008). Also unlike the UK, direct forms of age discrimination – such as age-based wage increase systems – have been accepted as legitimate means to achieve business objectives such as retaining skilled workers (Watanabe, 2008). Employers were permitted to set a mandatory retirement age of fifty-five up until 1994 when it rose to sixty. It is due to rise to sixty-five by 2013 (Wood *et al.*, 2010).

Another important characteristic of the institution of *lifetime employment* is its gendered nature: this institution has characteristically been experienced and thus reproduced by male workers (Brinton, 1994). As a predominantly male-centred labour market institution, lifetime employment has long rendered the labour market of Japan a distinctively gendered institution, from which female workers are often excluded (Abe, 2011).

Generally, these Japanese-style employment institutions have rendered older workers a very costly human resource to maintain, particularly after they reach mandatory retirement ages (Wood *et al.*, 2010). For instance, a national employer survey conducted by the Japanese Ministry of Labor and Welfare (MHLW) in 2008 suggests that 42 per cent of employers across the industrial sector, and regardless of company size, reported that it was financially burdensome and challenging to their HRM practices to continually employ older workers beyond their mandatory retirement ages (mostly age sixty). Of those employers who reported that they continued employment of older workers beyond mandatory retirement ages, 67 per cent reported that they needed financial assistance from the government in order to practice post-mandatory retirement age employment (MHLW, 2010). According to a survey conducted in 2005, 69 per cent of employers in Japan reported that they had explicitly posted upper age limits when hiring (MHLW, 2009). However, the majority of employers in Japan also reported concerns about potential problems in losing older workers at their workplaces. In 2007, 82 per cent of employers reported that it would be a problem to lose older employees; and 48 per cent of them felt that the main problem was the transmission of job skills and knowledge to younger employees (MHLW, 2010).

There is therefore an apparent demand for older workers within Japanese workplaces, but a concern about the affordability of retaining them. A representative from AEPA argued:

[Retaining senior employees] is a huge dilemma for many employers in Japan today, particularly large-scale organisations . . . they needed to find ways to retain seasoned skills and experience-based knowledge that many older workers could offer to them . . . [but] it is very expensive for many employers to retain them as regular employees.’ (AEPA)

Employers tackle these problems through two means. First, older workers who are post-mandatory retirement age may be transferred to another post or a subsidiary and employed on a new contract of employment, usually on lower pay with less job responsibility (Casey, 2005). This is known as the processes of ‘Shukko and Tenseki’: temporary and permanent transfers within and between organisations (Sato, 1996). Second, there are both pension incentives and state subsidies to older workers to make up for wage losses resulting from job change at or around the mandatory retirement age (Takayama, 2001).

Since the mid-1960s, the government has implemented a pension-related policy that ultimately aims to protect and promote employment opportunities for older workers. In 1965, the government activated the *Old-Age Working Pensioner Scheme*, which, generally, aimed to allow old-age pension beneficiaries to continually remain in the labour market in order to meet his or her economic needs (OECD, 2004). Similar to the UK, Japanese state pension provision is amongst the lowest in the developed world, replacing 41 per cent of average earnings (OECD, 2011a). In other words, since this 1965 policy, the government in Japan has generated a large number of working pensioners (Yamada and Higo, 2011). Income from work is therefore an important part of retirement income.

Unlike in the UK, in Japan mandatory retirement rules are still accepted. However, public policies have imposed requirements on employers to gradually increase older workers’ employment rates. Prior to 1986, there was a duty on employers to ensure that at least 6 per cent of their employees were aged fifty-five plus, but these regulations did not function well because the law was not compulsory. As a result, government changed its focus from monitoring the employment rates of older workers to regulating the mandatory retirement age policies of employers (Yamada and Higo, 2011). An officer at MHLW states:

Since the early 1980s, we [MHLW] have been fully aware of the unprecedented rate of population ageing that the country has been experiencing . . . Of course, employers don't want us to intervene in their business [corporate mandatory retirement policies]. But, to us, direct regulation of the mandatory retirement age was simply necessary for the country's future. (MHLW)

Since 1986, the government has aimed to prolong older workers’ working lives as much as possible (Williamson and Higo, 2009). Policy makers in Japan do so by intervening in the labour market with two major administrative strategies: mandating that employers gradually increase the age criteria set for mandatory retirement rules, and continuously develop active labour market programmes designed specifically to promote older workers’ labour force participation.

In 1986, the 1971 *Special Measure Law* was amended to the *Laws Concerning the Stability of Employment Opportunities for Older Persons* (the *1986 Law*), which has been continuously revised and amended to date as the basic legislative framework within which government policy makers develop and implement administrative measures to support older workers’ labour force participation (Seike and Yamada, 2004; OECD, 2004). Under the enactment of the *1986 Law*, employers were not only required to be prepared but also to propose specific measures for supporting their older employees’ prospective employment to the government, labour unions and employees (Higo and Yamada, 2009).

Through the 1994 partial revision of the *1986 Law*, the government mandated that employers set the minimum mandatory retirement age at sixty or older, and simultaneously issued administrative guidance for employers to endeavour to reform existing workplace policies and practices so as to prepare for further increasing the minimum age to sixty-five (MHLW, 2010). At around the same time, the government announced its future administrative plan to gradually increase the minimum eligibility age for part of the public pension benefits (Seike and Yamada, 2004). Japan's public pension scheme is two-tiered, and the eligibility age for the benefits of each tier has been set and increased separately. The second-tier of the public pension benefit, the earnings-related benefit, becomes available to workers when they reach the mandatory retirement age (usually age sixty in 1994). In 1994, the government announced plans to increase the minimum eligibility age from sixty to sixty-five for the benefit from the first-tier pension, which is financed with a flat-rate contribution from all citizens in the country. In 2001, furthermore, the government also announced plans to reduce the benefits from this component of the pension program and to increase payroll tax contributions from the beneficiaries to this program (Seike and Yamada, 2004). Further, in 2005, a revision of the pension laws removed a clawback of up to 80 per cent against pension recipients who simultaneously draw wages (OECD, 2012).

Finally, the Japanese government established a network of employment centres to support the older unemployed back into work. Since a 2002 amendment to the *1986 Law*, the Silver Human Resource Centres (SHRC) have evolved, under the slogan of ‘active aging society’, into a comprehensive public welfare programme for older citizens (Cabinet Office, Government of Japan, 2007). These were developed not only to help older job seeker find employment opportunities, but also to allocate resources for social networking and community integration by linking them with non-profit recreational and community service programmes available nationwide. Established in 1986, the number of the SHRCs has steadily increased from 425 in 1989 to 1,672 as of 2012 in parallel with the increase in the level of funding for the programme from USD 580 million in 1989 to USD 3.1 billion as of 2012 (National SHRC Corporation, 2013). Six per cent of older workers aged sixty-five to sixty-nine found their jobs via SHRC (Williamson and Higo, 2009). Furthermore, as a representative of SHRC notes:

Since . . . 2003, SHRC chapters have started providing registered members with a variety of free-of-charge job-training services . . .We have been providing these special services in collaboration with a number of business owners’ associations and other public employment security agencies, such as *Harowaku*, which manages social benefits for unemployed workers. (SHRC)

As part of its effort to support older workers’ labour force participation, since 2004 the government has been providing employers with subsidy programmes as financial incentives for them to continue employing their older workers at least until age sixty-five (MHLW, 2010). Employers are now required to fulfil at least one of the following three measures: (1) fully abolish mandatory retirement rules, (2) set the minimum age for mandatory retirement at sixty-five or older, or (3) introduce employment policies aimed to continue employing older workers until at least age sixty-five (MHLW, 2009). Few employers have abolished mandatory retirement rules primarily due to older workers’ high wages relative to what employers perceive as their actual productivity at work (Seike and Yamada, 2004). With the second option, employers would have to continue to employ older workers without changing their employment status, job content or wages until at least age sixty-five. The third option does not mandate that employers unconditionally guarantee secure employment to older workers until age sixty-five. Rather it merely requests that employers introduce measures that are aimed to provide their older workers with opportunities to remain employed at least until age sixty-five. In balancing the pressure to reduce costs associated with human resources and the requirements of the latest revision of the *1986 Law*, most employers in Japan have chosen the last option and have re-employed workers who have reached age sixty in temporary or part-time positions with reduced wages and responsibilities (MHLW, 2010). Furthermore, despite employers maintaining mandatory retirement, the government has recently expanded the administrative scope of its intervention in the country's ageing labour market. Since 2006, the government has begun a series of national campaigns aimed at encouraging employers to retain employees up to age seventy (Higo and Klassen, 2014). For instance, the government has implemented award programmes that provide grants for employers who introduce corporate policies to this end. In these programmes, the government publicises the names of these ‘model employers’, whom the government recognises as being well prepared for the rapid ageing of the country's workforce to encourage other employers to emulate their practices (MHLW 2010).

## Discussion

As Ebbinghaus (2001) argues, in developed countries, in Western Europe in particular, early retirement routes have been the product of collusion between the state and businesses. These routes are created primarily in order to ease mass unemployment during periods of economic downturn, maintain peaceful labour relations and shed high-cost older workforces.

Earlier, we argued that the government in both countries might be acting as an IE with the goal of reversing the collusion toward early retirement in favour of a working pensioners model. The evidence that this paper presented demonstrates that the institutional and social contexts of the status quo within the two countries are both similar and resistant to an extended working life. Although Japanese employers do provide work opportunities to older workers, they are as reluctant to do so as their British equivalents. At the same time, while UK workers retire earlier than their Japanese counterparts, they would be willing to delay retirement if offered equivalent opportunities to phase into retirement. Healthy life expectancy does not sufficiently explain the differences in work longevity between Japan and the UK. Japanese women live significantly longer healthier lives than their UK counterparts, but it is the Japanese men who are working to a later retirement age. The observed divergence in HRM practices in relation to older workers would therefore suggest that the government does play a role in shaping organisational age management practices, with the Japanese government particularly successful in compelling employers to create work opportunities for older workers. However, as Battilana (2006) observed, the government is constrained by the institutional systems which it has also replicated. In this case, the UK government is constrained by a history of a state condoned early retirement culture. We next consider the effectiveness of the government in the two countries in championing a working pensioners HRM model. Judging simply by labour force participation rates in the two countries, the evidence would suggest that Japan has been more effective in keeping older people economically active, despite maintaining mandatory retirement. Until 2013, the mandatory retirement age in Japan was lower than the state pension age, with the expectation that employers would provide post-retirement work opportunities for older workers.

Although both governments have adopted light-touch approaches to age issues, we have highlighted two sets of measures which differentiate their approaches. First, the Japanese government has made employers’ retention of mandatory retirement policies contingent upon the achievement of older workforce participation targets. The UK, meanwhile, has adopted a strategy of persuading employers to adopt age positive practices, and the present Coalition government has largely deferred to non-government organisations (NGOs) in making this case. Second, the Japanese government has invested heavily in job placement support for workers over the mandatory retirement age. Such workplace and individual specific interventions would be difficult to implement in the liberal UK system, particularly as business pressure is directed toward earlier rather than later retirement. Although the Japanese model of supporting the older unemployed may be used by the UK opposition Labour party as a model if it returns to power in the near future, when it was in government only modest resources were used in this way. Similarly, the present government has also devoted few resources to finding employment for older displaced workers.

The UK approach, based on the ‘business case’ for extending working lives, has not enabled the UK government to act as an IE. This is because the approach can be considered reactionary to the economic climate in the UK at the time. A recent survey of employers indicates that a majority favoured a re-introduction of the DRA (Irwin and Mitchell, 2012). Further, the government itself has largely relied on early retirement to manage job attrition. UK governments, hence, have not attempted to change existing institutions, but have implemented policies that have reacted to the situation generated by organisations. This is even though the UK's institutional field of labour market regulation with regard to older workers is considered to be institutionalised to a lesser degree than that of Japan. As noted by Battilana *et al.* (2009: 75), the low degree of institutionalisation in a given field might ‘provide opportunities for strategic action’. The UK governments were therefore expected to have more leeway to push policy agendas that lead to institutional change, which, based on our analysis, UK governments did not do to the extent to which Japanese governments triggered institutional change.

## Conclusion

Our study of older workers generally confirms Nasra and Dacin's (2010) proposition that governments can and do exploit opportunities for entrepreneurial action. Indeed, Ebbinghaus (2001) argued that successive governments within Europe worked in tandem with other institutional actors (trade unions and employers) in preserving the culture of early retirement which was seen to benefit each party. However, the government as an IE is uniquely both constrained and enabled by the institutional role of the state.

For the UK, the limitation is fairly straightforward. Successive governments have been self-limited by the goal of being the least regulated European economy. The UK government has been able to pursue older workforce participation as a social goal, both in terms of implementing age discrimination regulations and abolishing the DRA. These measures are in keeping with the role of the regulatory state in using the levers of due process to pursue normative goals such as equality in the workplace context (Edelman, 1990). This would suggest the following: first, although Japan has been posited by the UK opposition party as a model for government in facilitating extended working lives, its adaptation in the UK needs to take account of Japan's productivist welfare state and developmentalist business system which facilitates governments in pursuing long-term strategies in managing changing age demographics. Second, by contrast, UK governments are compelled to prioritise short-term goals such as tackling youth unemployment and public sector job cuts.

Further, in the case of Japan, the government has been enabled to a greater extent than that of the UK to coerce employers, and leverage the welfare system in pursuit of creating work opportunities for older people. However, its actions are limited to economic goals. Indeed, it has been noted in relation to gender equality that economic conditions, and the role of women as a contingent workforce, have largely set the pace and direction of public policy (Lee and Fujita, 2011).

Finally, we would argue that the role of the government as an IE in an HRM context deserves further exploration. This is because government action can simultaneously replicate and challenge existing institutional norms. With regard to retirement, pension and employment rules can run counter to governments’ work and retirement objectives, and short-term pressures (such as youth unemployment) can force a policy reversal (as observed in the UK). Further exploration in this area, particularly from an East–West perspective could cast more light on how the state can adapt to long-term age demographic challenges.

## Notes

1 In Japan, however, there is a greater proportion in the labour market as there is a greater share of workers classed as ILO unemployed.

2 Seldon v Clarkson.

3 Figures from DWP Statistical Summary.
